# An Enzoötic Attack of Chorea among Cattle1Read before the Tenth Annual Meeting of the Missouri Veterinary Medical Association, October 22 and 23, 1901.

**Published:** 1902-01

**Authors:** A. D. Knowles

**Affiliations:** Nevada, Mo.


### REPORTS OF CASES.

### AN ENZOÖTIC ATTACK OF CHOREA AMONG CATTLE.[1]

By A. D. Knowles, V.S.,
NEVADA, MO.

About September 20, 1900, I was on the farm of a Mr. Arnold, vaccinating some cattle, and my attention was called to a grade-polled Angus heifer about a year and a half old; she stood among a bunch of cattle on the opposite side of the lot, with her head turned toward us; the head was moving from side to side and in a rotary motion; the eyes staring; the front feet extended, as if to prevent falling forward; the hind legs also placed in a bracing position, and the animal was very nervous.

When I approached her she started excitedly, stepping high, the front feet almost touching her ears, and after going a few steps she fell on her side in a state of eclampsia. I approached and found her trembling, with eyes rolled back; respiration and pulse very rapid. I did not take her temperature at that time, but found on later examinations that the temperature was elevated from one to three degrees. After leaving her for a few minutes, she arose and passed along the opposite side of the lot with the same unsteady gait, frequently falling forward.

I was informed by Mr. Arnold that he had had cattle affected like her for four previous years, that it usually made its appear-

[1] Read before the Tenth Annual Meeting of the Missouri Veterinary Medical Association, October 22 and 23, 1901.

ance in October and lasted several weeks ; and that he had never lost any showing the symptoms; and said he had had the services of veterinarians before, but that they had failed to successfully treat the affection.

I was on this farm several times during the autumn and winter, and saw about seventy-five head of his herd of two hundred and fifty cattle show the symptoms just described ; the affected cattle would continue to eat and thrive, but would frequently fall, while suffering from the spasms, into ditch and creek, and be unable to arise until helped.

The only one which Mr. Arnold has lost while showing the symptoms was a steer about a year old, which had fallen into a ditch on the night of January 3, 1901 ; everything indicated that the animal chilled to death while lying in the ditch on his back.

I held an autopsy about thirty-six hours after death, and found the following conditions present: The body showed no signs of decomposition and there was no tympanitis, the post-mortem was held by the field operation, exposing all of the internal viscera as a whole, after which the contents of the intestines, stomach, and bladder were exposed ; then we examined carefully the external and internal conditions of the heart, lungs, liver, kidneys, and spleen. There was nothing to indicate disease or abnormality until the cranial cavity was reached. Upon opening that cavity the dura mater and arachnoid showed nothing characteristic, but there seemed to be much more than the normal quantity of the subarachnoid fluid, and the vessels of the pia mater were greatly distended with blood; there was nothing else to attract especial attention.

There were a greater number of cattle affected on this farm last year than there had been any previous year. The owner has had sheep which, he says, have showed the same symptoms, and he thinks some have died from the affection. The farm referred to lies in the northern part of Vernon County, Missouri, and is composed of about eight hundred acres of black lime-stone soil ; it is all set in timothy, clover, and English blue grass. The farm is exceptionally clear of weeds and brush except forty acres, of which I shall speak later; a creek runs through the farm from south to north, but the cattle receive water mostly from tanks fed by large ponds, and the water-supply in the tanks is regulated by floating valves; the ponds are all fenced in so that no stock can get to them. The pasture was excellent, and the cattle did not receive any grain or hay until after January 1st, except two carloads of two-year old steers, which were being fed for the market; but those receiving no grain remained in splendid condition.

The forty acres referred to is fenced to itself and is not used during the summer, but is allowed to grow up in brush and weeds, and is used as a wind-break in winter. On one occasion about seventy-five head of cows and young cattle got into this enclosure, and within three days about twenty head were showing the symptoms; some were not able to stand for a few days, and others had to be pulled from ditches or creeks occasionally. During the time these cattle were so badly affected they continued to eat and digest their food. The cattle were removed from that pasture, and some of the affected ones improved rapidly, while others continued in about the same condition for several months.

On March 22, 1901, I accompanied our State veterinarian to this farm. There were several cattle still affected, and as thorough an investigation as could be made in one day was made by Dr. Luckey. Plans were laid to gain more knowledge of the affection; but as the grass came on the cattle gradually grew better, until by May 1st there was not an animal showing any perceptible symptoms.

The grade-polled Angus heifer I spoke of as being first affected did not make permanent improvement until spring; but, like all of the others, she showed signs of improvement, to be succeeded by periods of the most aggravated symptoms. After the grass was good in the spring she gradually regained her normal actions, and was shipped to market about the middle of the summer, apparently having made as good a growth as though she had never been affected.

There was no treatment recommended for the cattle, and none was given except that Mr. Arnold, on his own judgment, gave treatment to two cattle similarly affected. One of them, receiving full doses of iodide of potassium, and the other one full doses of the bromide, did not show any change of symptoms. There were other cattle in the same neighborhood affected and showed the same symptoms.

Mr. Arnold has his cattle graded, and keeps each grade in a pasture to itself; and a very noticeable fact is that cattle that were on pasture which was used for meadow—*i. e.*, which was mowed and then pastured after the grass came up sufficiently—did not show any of the above symptoms while they were confined on such pasture, but when moved from that on to land which had been used for pasture the entire season they quickly developed the symptoms.

Cattle of all ages were affected; also some of those on full feed of grain, but those on grain were also on grass.

The symptoms developed earlier in the year 1900 and lasted longer than previous years. Mr. Arnold has had no cattle affected yet this year—October 15th.

I hope this contribution will stimulate a discussion which will give new thoughts and more knowledge on the subject.

